# Whole-Transcriptome Sequence Analysis of *Verbena bonariensis* in Response to Drought Stress

**DOI:** 10.3390/ijms19061751

**Published:** 2018-06-13

**Authors:** Bei Wang, Xue-Qi Lv, Ling He, Qian Zhao, Mao-Sheng Xu, Lei Zhang, Yin Jia, Fan Zhang, Feng-Luan Liu, Qing-Lin Liu

**Affiliations:** 1 Department of Ornamental Horticulture, Sichuan Agricultural University, 211 Huimin Road, Wenjiang District, Chengdu 611130, Sichuan, China; s20167108@stu.sicau.edu.cn (B.W.); 20150853@stu.sicau.edu.cn (X.-Q.L.); s20162113@stu.sicau.edu.cn (L.H.); s20167109@stu.sicau.edu.cn (Q.Z.); 20150782@stu.sicau.edu.cn (M.-S.X.); 14069@sicau.edu.cn (L.Z.); 13864@sicau.edu.cn (Y.J.); 13305@sicau.edu.cn (F.Z.); 2 Shanghai Key Laboratory of Plant Functional Genomics and Resources, Shanghai Chenshan Plant Science Research Center, The Chinese Academy of Science, Shanghai Chenshan Botanical Garden, 3888 Huagong Road, Songjiang District, Shanghai 201602, China

**Keywords:** *Verbena bonariensis*, drought stress, transcriptome sequencing, differentially expressed genes

## Abstract

Drought is an important abiotic factor that threatens the growth and development of plants. *Verbena bonariensis* is a widely used landscape plant with a very high ornamental value. We found that Verbena has drought tolerance in production practice, so in order to delve into its mechanism of drought resistance and screen out its drought-resistance genes, we used the RNA-Seq platform to perform a de novo transcriptome assembly to analyze Verbena transcription response to drought stress. By high-throughput sequencing with Illumina Hiseq Xten, a total of 44.59 Gb clean data was obtained from T01 (control group) and T02 (drought experiment group). After assembly, 111,313 unigenes were obtained, and 53,757 of them were annotated by compared databases. In this study, 4829 differentially expressed genes were obtained, of which 4165 were annotated. We performed GO (Gene Ontology) and KEGG (Kyoto Encyclopedia of Genes and Genomes) pathway enrichment analyses, and explored a lot of differently expressed genes related to plant energy production, hormone synthesis, cell signal transduction, and metabolism to understand the stress response of Verbena in drought stress. In addition, we also found that a series of TFs related to drought-resistance of Verbena and provide excellent genetic resources for improving the drought tolerance of crops.

## 1. Introduction

Adverse environmental factors such as low temperature and salt stress, together with drought, prevent plants from realizing their full genetic potential and have been the main problems facing agriculture [1]. Drought stress affects plant growth and development by affecting the plant respiration, growth, photosynthesis, assimilate partitioning, moisture and nutrient relationships, and drought-induced crop yield losses may outweigh losses from all other causes [2]. A series of physiological and biochemical reactions of the plant under drought conditions are altered through gene regulation, such as activation of respiration, repression of cell growth and photosynthesis, and stomatal closure [3]. Accelerating the pace of revealing drought-tolerance mechanisms will greatly help traditional breeding efforts and the application of modern genetic methods in improving the drought tolerance of crops [4].

Many of Verbenaceae’s plants have important medicinal values, such as *Aloysia triphylla* [5] and *Cordia verbenace* [6]. Verbena (*Verbena bonariensis* L.) is an excellent ornamental landscape plant with extensive management. It has a flourishing and long flowering period, such that it plays an extremely important role in landscape layout. In addition to its ornamental value, Verbena has shown good performance under drought stress in production practice, which provides new thinking for our study on plants’ responses to abiotic stress. However, the understanding of its drought-resistance mechanism is still in the early stage. Presently, high-throughput sequencing technology has been widely used to reveal plants’ intrinsic physiological mechanism at the molecular level, such as model plants *Nicotiana tabacum* L. [7,8,9], *Oryza sativa* L. [10,11,12], and *A. thaliana* L. [13], as well as others, like *Glycine max* (Linn.) Merr. [14], *Brassica napus* L. [15], and *Cucumis sativus* L. [16], for its high accuracy and sensitivity of gene discovery. However, in non-model plants, the progress of drought-resistance research has been slow and unevenly developed, and is thus in need of a lot of effort. In this study, we analyzed the transcriptome and differently expressed genes of Verbena by using high-throughput sequencing technology, ultimately analyzing its mechanism of drought resistance and providing potential drought resistance gene information for resistance breeding work.

## 2. Results

### 2.1. Phenotypic and Physiological Indicators of Verbena under Drought Stress

**Figure 1:**
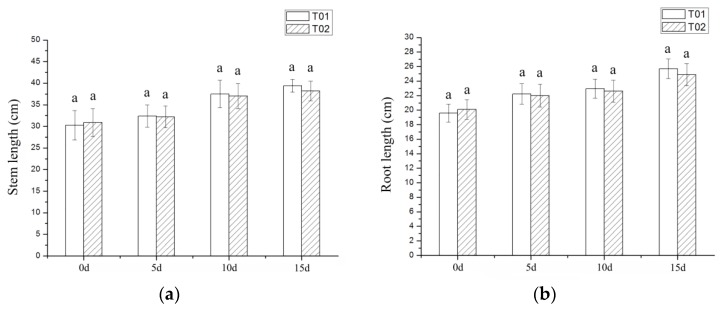
Morphological indexes of Verbena under drought stress. T01 is the control group and T02 is the drought experiment group: (**a**) The determination of stem length; (**b**) The determination of root length.

**Figure 2:**
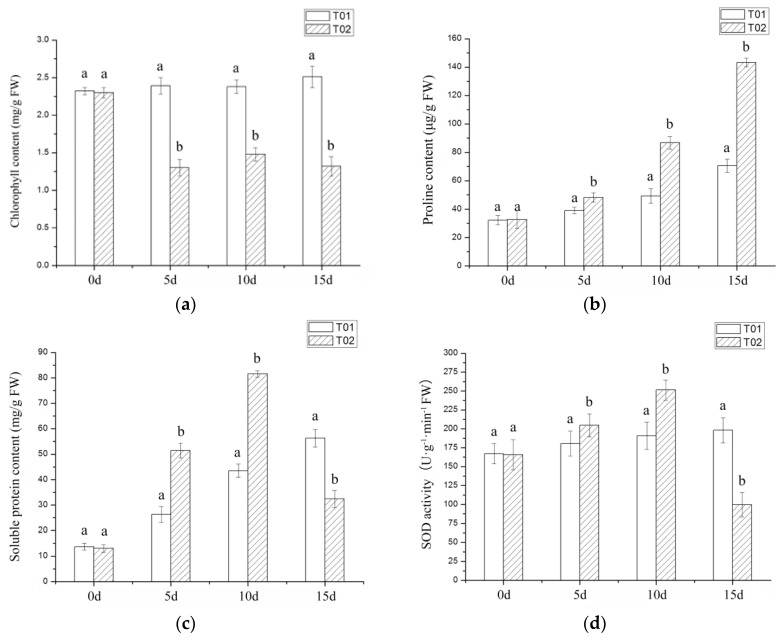
Physiological indexes of Verbena under drought stress. T01 is the control group and T02 is the drought experiment group: (**a**) The content of chlorophyll; (**b**) The content of Pro; (**c**) The content of soluble protein; (**d**) The content of SOD.

**Figure 3:**
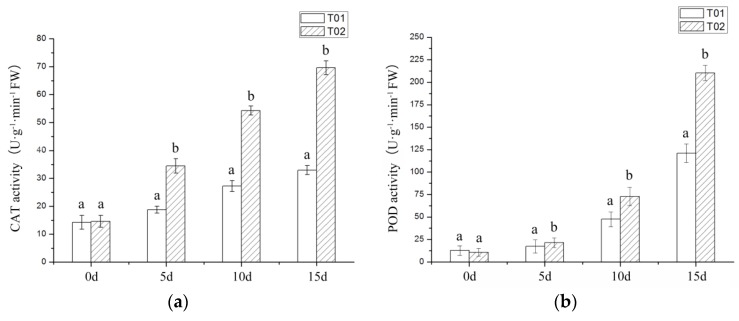

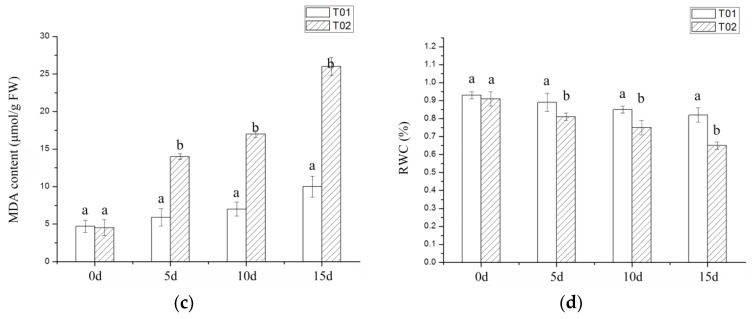
Physiological indexes of Verbena under drought stress. T01 is the control group and T02 is the drought experiment group: (**a**) The activity of CAT; (**b**) The activity of POD; (**c**) The activity of MDA; (**d**) The relative water content of leaves.

In this study, the morphology of Verbena plants was not significantly affected by drought stress (Figure 1a,b). Different from the morphological indicators, physiological indicators of Verbena had undergone significant changes. The chlorophyll content of leaves first decreased rapidly and then increased by a small margin (Figure 2a), the content of proline (Pro) and soluble protein showed a trend of increasing (Figure 2b,c), the content of superoxide dismutase (SOD) reached the highest level on the 10th day, both catalase (CAT) and peroxidase (POD) gradually increased (Figure 2d and Figure 3a,b), malonaldehyde (MDA) content also increased and relative water content (RWC) did not significantly decrease (Figure 3c,d).

### 2.2. Sequencing and Annotation of Transcription and Unigenes

Based on Sequencing By Synthesis (SBS), six transcriptomes were sequenced by Illumina Hiseq Xten (Illumina, CA, USA). We obtained a total of 44.59 Gb clean data, and in each sample, the Q30 base was not less than 92.87%, the CG (guanine and cytosine basic groups) content was not less than 44.41% (Table SA1). The Pearson’s Correlation Coeffiencient *r* between T1–T3 and T4–T6 was listed in Figure SA1. Using the de novo assembly program Trinity [17] to assemble short-reads, a total of 258,326 transcripts with an average length of 1139.68 bp were obtained. After continuing to cluster and assemble the transcripts for analysis and a total of 111,313 unigenes with an average length of 697.08 bp and N50 of 1223 bp were obtained, among which 40,340 (36.24%) were over 500 bp in length (Table SA2 and Figure SA2).

To predict and analyze the function of the Verbena unigenes, we used BLAST software to compare the amino acid sequence with NR (NCBI non-redundant), KOG (euKaryotic Orthologous Groups), COG (Clusters of Orthologous Groups), GO (Gene Ontology), KEGG (Kyoto Encyclopedia of Genes and Genomes), Pfam (Protein family) and Swissprot-Annotation (a manually annotated and reviewed protein sequence database) database, setting BLAST parameters E-value ≤ 1 × 10^−^^5^ and HMMER parameters E-value ≤ 1 × 10^−^^10^ as standard, a total of 53,757 unigenes was obtained, accounting for 48.29% (111,313) of the total. There were 51,352 (95.53%), 28,994 (53.54%), 14,836 (27.60%), 16,938 (31.51%), 20,988 (39.04%), 32,735 (60.89%) and 27,990 (54.53%), unigenes assigned to these databases, respectively (Table SA3). Only 48.29% of unigenes can be matched to known genes, which may be caused by the current lack of studies on Verbena.

A total of 18,104 (35.27%) of unigenes were annotated to *Sesamum indicum*, which means that *Sesamum indicum* had the highest level of homology with *Verbena*, followed by *Erythranthe guttata* (8.60%) and *Erysiphe necator* (7.56%). In addition, *Verbena* and *Sesamum indicum* are quite similar in morphology because they have spike inflorenscence, which is morphological proof of their high homology (Figure SA3).

There are 28,994 unigenes assigned to KOG database and 14,836 to COG database. In these two databases, the proportion of “Signal transduction mechanisms” related to plant resistance respectively occupied 9.85% and 9.45%. In addition, the amount of unigenes assigned to the classes related to plants’ response to stress, such as “Defense mechanisms”, “Secondary metabolites biosynthesis, transport and catabolism” and “Inorganic ion transport and metabolism”, was 7.40% and 10.50% in the two databases, respectively (Figure SA4).

20,988 unigenes were annotated and classified into 3 categories of GO: cell component (CC), molecular function (MF), and biological processes (BP). Most of the genes were assigned to the biological process (59.16%), followed by the molecular function (23.87%) and cellular component (16.97%) (Figure SA5). Among these, the first three categories of BP were “metabolic process” (2.85%), “cellular process” (2.35%) and “single-organism process” (1.84%).

KEGG is a suite of databases and associated software for understanding and simulating higher-order functional behaviors of cells or the organisms based on their genome information. There were 16,938 (31.51%) unigenes allocated to 129 pathways of KEGG, and the pathways assigned with the most genes are “ribosome” (922), “carbon metabolism” (708) and “biosynthesis of amino acids” (622).

### 2.3. Analysis of Differentially Expressed Genes (DEGs)

Using Bowtie [18], the clean reads were compared to the unigene library. Genes’ expression levels were estimated by RSEM (RNA-Seq by Expectation Maximization) [19], and according to the results, the expression abundance of a single gene was expressed as the value of FPKM (transcript fragment per million fragments). The volcano map showed that there were 4829 DEGs, where 3841 (79.54%) were up-regulated and 998 (20.46%) were down-regulated (Figure SA6). Finally, 4165 (85.72%) DEGs were annotated, most of which were annotated to NR (4155) and eggNOG (3957), followed by Pfam (3419), Swissprot (2577), KOG (2426), GO (1756), COG (1554) databases and KEGG (1521) (Table SA3). Clustering results of all DEGs are shown in Figure SA7.

**Figure 4:**
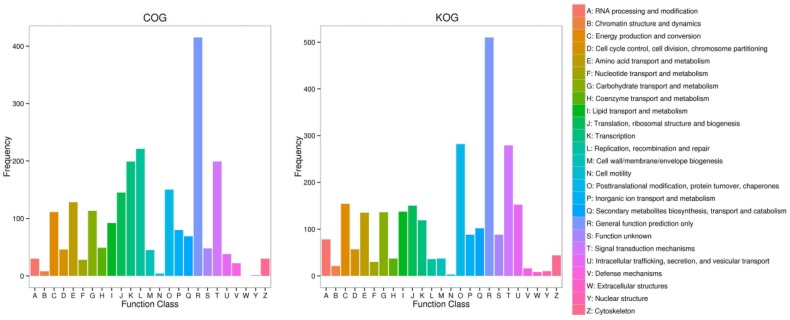
The bar chart of unigenes’ functional classification annotated in COG and KOG databases. The abscissa is the function classifications of COG and KOG databases and the ordinate is the number of DEGs annotated in it.

Among the DEGs assigned to KOG and COG, the number of genes in these classes related to plant-resistance of abiotic stress such as “inorganic ion transport and metabolism”, “secondary metabolites biosynthesis, transport and catabolism” and “defense mechanisms” were, respectively, 206 (8.50%) and 171 (11.00%), higher than it was for unigenes (Figure 4).

**Figure 5:**
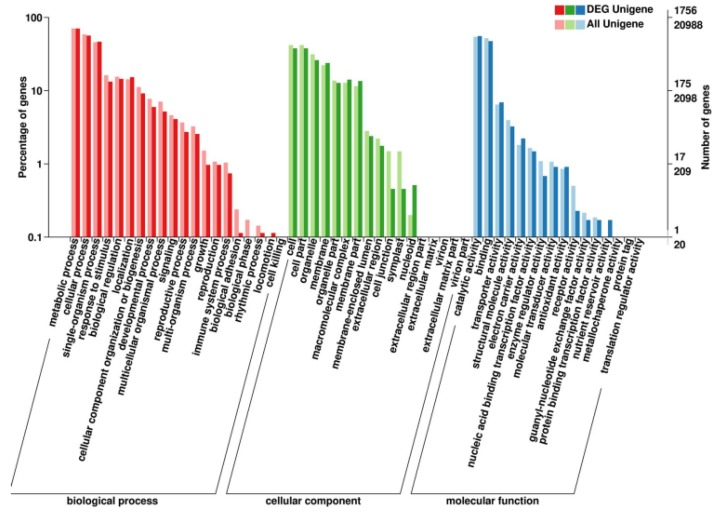
The bar chart of DEGs annotated in the GO classification. The ordinate at the left represents the percentage of the number of genes, the right ordinate represents the number of genes. The above of two ordinates is the number of DEGs, the following is the number of all genes. The abscissa is the classification of GO. The dark bar represents the number and proportion of DEGs that are enriched in GO function, and the light bar represents the number and proportion of genes that are enriched for each GO function.

We found that BP, MF and CC accounted for 58.48%, 24.64% and 16.88% respectively by the analysis of 1756 DEGs in GO database. In the entire genetic background and DEGs, the enrichment of genes for each node in the GO database is listed in Figure 5. The top three classes in BP are “DNA integration”, “RNA-dependent DNA replication”, and “photosynthesis, light harvesting”, the most notably of which is the enrichment of “photosynthesis, light harvesting”, and the most noticeable of which in MF are the “acyl-CoA dehydrogenase activity” and “oxidoreductase activity, acting on paired donors, with oxidation of a pair of donors resulting in the reduction of molecular”; the *p*-values obtained by the KS test were 0.00024 and 0.00016, respectively. “Ribosome”, “fungal-type vacuole membrane” and “nucleus” are the top three in CC, followed up by “photosystem II” and “photosystem I”, with *p*-values of 8.3 × 10^−^^5^ and 1.4 × 10^−^^4^. The top five significantly enriched nodes of BP, MF and CC are listed in Table SA4.

The DEGs were distributed to 126 lower pathways of KEGG. The first three pathways with the highest enrichment factor were “photosynthesis antenna proteins”, “betalain biosynthesis” and “flavone and flavonol biosynthesis”, with factors of 6.84, 6.36 and 4.71, respectively. The top ten most significantly enriched pathways of DEGs in KEGG are listed in Table SA5 . In the “Photosynthesis antenna proteins” pathway, the class with the highest enrichment factor had a total of 20 DEGs, which were all annotated to the homologous gene annotations of “Light-harvesting Chlorophyll a/b Binding (LHC) Proteins”—the apoproteins of the light-harvesting complex of photosystem II (PSII) [20].

### 2.4. DEGs of Transcription Factors (TFs) under Drought Stress

**Figure 6:**
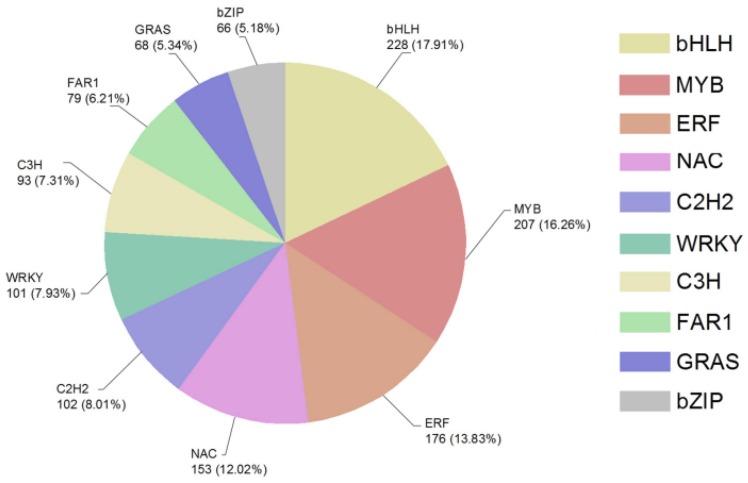
The sector diagram of TFs’ classification and number in DEGs in response to drought stress.

In this study, a total of 2146 (44.44%) transcription factor DEGs were identified, of which 1656 (77.17%) were up-regulated and 490 (22.83%) were down-regulated. The TFs mainly focused on bHLH, MYB, ERF, NAC and C2H2 families, with 228 (10.62%), 207 (9.65%), 176 (8.20%), 153 (7.13%) and 102 (4.75%) DEGs (Figure 6), respectively. The number of up-regulated genes of the ten were 171 (75%), 157 (75.85%), 137 (77.84%), 122 (79.74%) and 76 (74.51), respectively.

### 2.5. Expression Level of DEGs’ Changes and Verification Using qRT-PCR

**Figure 7:**
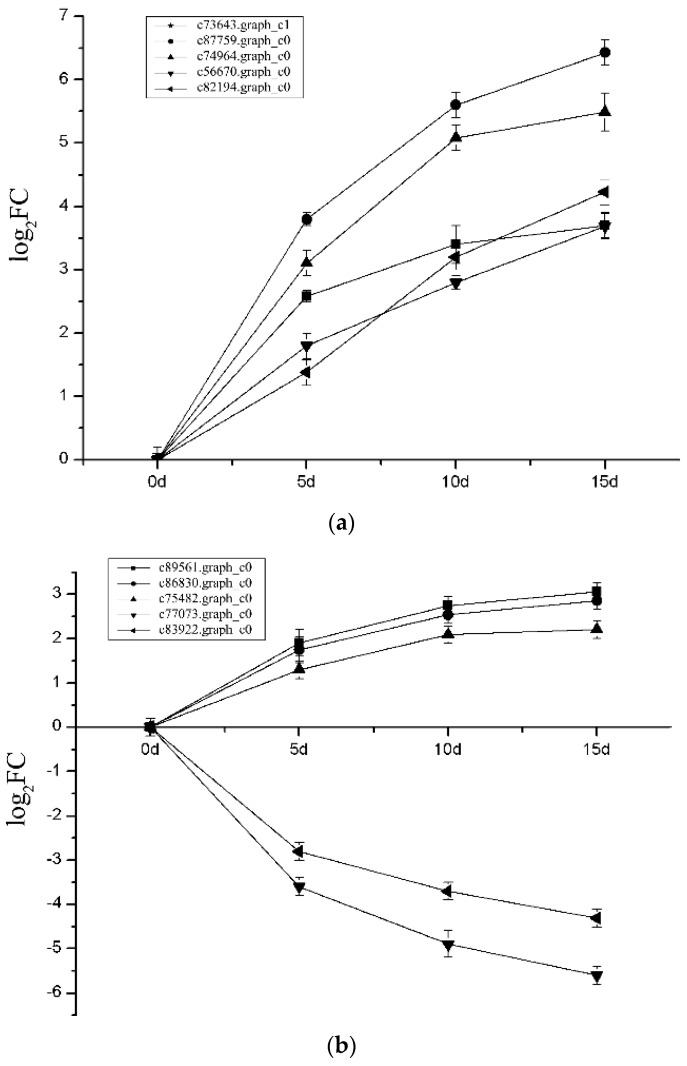
The line chart of the expression level of the 10 DEGs varied with the degree of drought during the experiment. (**a**) Five DEGs varied with the degree of drought during the experiment; (**b**) another five DEGs varied with the degree of drought during the experiment.

**Figure 8:**
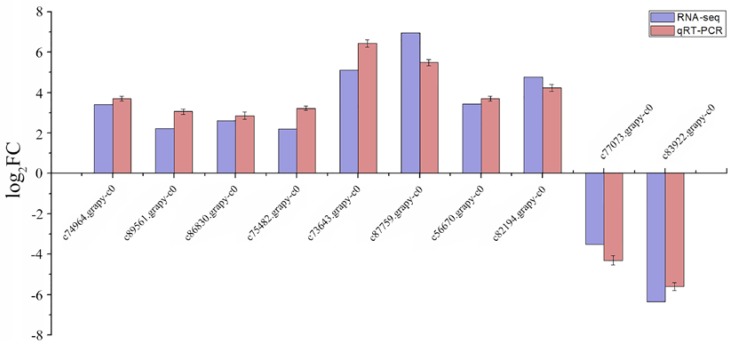
The bar chart of results of qRT-PCR in 15th day. The relative expression level of ten DEGs identified in the comparison between RNA-Seq and qRT-PCR. The genes relative expression level were determined by 2^−^^ΔΔ^*^C^*^t^ method.

We selected ten genes involved in different important biological processes to perform qRT-PCR, including genes related to “energy production and conversion”, “transcription factors”, “LHCB2 proteins”, “glutamine synthetase”, “protein kinases”, “lipid transport and metabolism”, “carbohydrate transport and metabolism” and “nitrogen metabolism”. During the experiment, similar to indexes of physiological, the expression level of these DEGs were all up-regulated with the increase of stress time (Figure 7a,b). To further verify the expression level of genes obtained from Illumina Hiseq Xten, we compared the data obtained from the 15th day with sequencing, and the results showed a strong correlation between the two (Figure 8).

## 3. Discussion

### 3.1. Morphological and Physiological Index Analysis

Among the ten most abundant nodes in the BP of GO database, the most notable one is “photosynthesis, light harvesting”. Many studies [21,22] have shown that water stress can change the plants’ chlorophyll content, which can indicate the sensitivity of plants to water stress and directly affect the photosynthetic yield. Some studies have shown that drought increases the chlorophyll content of plants’ leaves [23], while some other studies believe it would be gradually decreased [24]. In this study, the chlorophyll content decreased rapidly and then increased by a small margin. Although it is unclear what the mechanism of water stress on chlorophyll content is, the fact is that increasing the chlorophyll content will enhance plants’ endurance to survive in adversity, and it is a kind of ability by which plants can adapt to drought stress, indicating that the Verbena has a certain drought tolerance.

In this experiment, the Pro content, soluble protein content, antioxidant enzyme activity and the MDA content were all increased. Pro and soluble proteins are the most common osmotic pressure regulators in drought-stressed plants. Plants that overproduce Pro and soluble protein might acquire the ability to tolerate environmental stresses such as drought and high salinity [25]. There are a large number of antioxidants that can prevent or repair the damage caused by reactive oxygen species and regulate redox-sensitive signaling pathways, such as POD, SOD, CAT and so on [26]. The degree of membrane damage on the leaves can be demonstrated by the content of MDA, which gradually increased in this study. Under the circumstances of drought force, the more drought-tolerant the plants are, the slower the water content of the leaves decreases. Figure 3d shows that on day 15, the RWC of T02 (stress group) was about 16% lower than that of T01 (control group), which performed better than *Hordeum vulgare* L. [27], *Zea mays* L. [28] and *Glycine max* (Linn.) Merr. [29], indicating that the leaves of Verbena have a certain water retaining capacity.

### 3.2. The Enrichment and Pathway Analysis of DEGs in GO and KEGG Databases

The chlorophyll content decreased sharply in the early stage of drought stress, and eventually increased to a lower level than T01. The 16 DEGs related to “porphyrin and chlorophyll metabolism” are listed in Table 1, and the enzymes of porphyrin metabolism were almost all up-regulated, while the enzymes of chlorophyll metabolism were almost all down-regulated. Analyzing the genes involved in the regulation of stomatal closure by ABA (abscisic acid), we found that some of the genes’ expression levels of PYL and PP2C were significantly up-regulated (Table 2), and according to the analysis of the “carbon fixation in photosynthetic organisms” pathway, we found that a number of genes related to “C4-Dicarboxylic acid cycle and carbon fixation pathways in prokaryotes” process showed an upward trend. This indicates that under drought stress, the photosynthesis of Verbena may be mainly inhibited by stomatal closure, and at the same time it may provide an adequate carbon source for photosynthesis by enhancing the biological carbon sequestration pathway. The DEGs in “photosystem II” and “photosystem I” of CC also showed a high degree of enrichment, indicating that drought had a great impact on the photosynthesis of Verbena, which is the direction we should focus on. In MF, “acyl-CoA dehydrogenase” and “oxidoreductase” are obviously enriched. Previous study has shown that deficiencies of acyl-CoA dehydrogenases can lead to disorders of fatty acid oxidation, leading to life-threatening metabolic disorders [30]. It is known that drought stress will produce a large amount of reactive oxygen species (ROS) in plants and start the massive production of oxidoreductase such as SOD. Therefore, the increase of “acyl-CoA dehydrogenase” and “oxidoreductase” is of great significance for plants in responding to drought stress.

**Figure 9:**
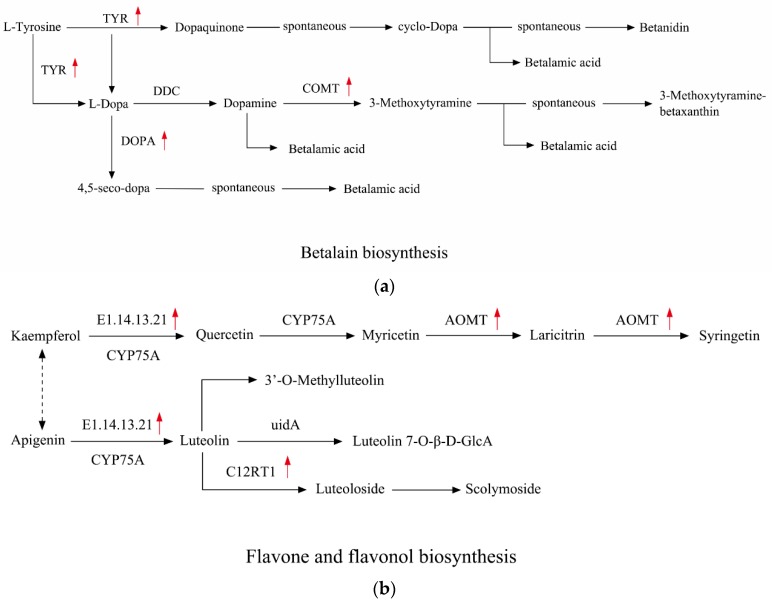
Two schematic pathway figures of “Betalain biosynthesis” and “Flavone and flavonol biosynthesis”: (**a**) Effect of drought stress on the expression of genes associated with betaine. (**b**) Luteolin and quercetin metabolism.

In the KEGG database, the first three pathways with the highest DEGs enrichment factors are “photosynthesis antenna proteins”, “betalain biosynthesis” and “flavone and flavonol biosynthesis”. After comparison, the homologous genes with almost all the down-regulated DEGs (Table 3) in “photosynthesis antenna proteins” pathway are related to LHC proteins, which may be involved in the drought resistance of plants and play an important role in crop environmental adaptability and yield [31]. Alberte R.S. et al. [32] showed that LHC proteins are a target easily attacked by water stress, and the loss of chlorophyll, the increase of chlorophyll a/b ratio and the decrease of photosynthetic unit under water stress are all caused by the decrease of LHC proteins. Vappaavuovi E. et al. [33] also confirmed that water stress reduced the LHC protein complex. In addition, some studies have showed that LHC proteins may be involved in the partial regulation of ABA signaling and play an active role in guarding cell signaling—these proteins may be induced by ABA and positive regulates ABA to inhibit stomatal opening [34,35]. Therefore, the decrease of LHC proteins will inhibit the production of ROS. However, due to the insufficiency research of LHC proteins, further research on its response to abiotic stress is needed. “Betalain biosynthesis” pathway has three distinctly up-regulated genes (Table 4). Betaine is the trimethyl derivative of the amino acid glycine, an efficient methyl donor that promotes fat metabolism and protein synthesis and the increasing of betaine biosynthesis has been shown to play an important role in osmoregulation of plants, which can help plants to withstand drought stress [36]. The “Flavone and flavonol biosynthesis” pathway has four distinctly up-regulated genes with an enrichment factor of 4.71, and studies have shown that the flavonoid substance may contribute to antioxidant functions in response to drought stress [37]. At present, there is still a lack of research on the changes in the content of secondary metabolites that have economic or medicinal value in Verbena under abiotic stress, but according to the schematic pathway figures of betalain and flavone (Figure 9a,b), we can preliminarily speculate that the content of flavonoids and betaine were up-regulated under drought conditions, while other plants also have similar research conclusions. Xing W. et al. [38] have shown that under drought stress, the endogenous leaf glycine betaine level of *A. thaliana* L. increased about 18-fold over that in the control plants, and similar results were also found in Pyrus bretschneideri Rehd [39] and *Hordeum vulgare* L. [40]. From the pathway of “flavone and flavonol biosynthese”, we can see that all DEGs are up-regulated (Table 5) and there are two kinds of useful downstream production—luteolin and quercetin. Studies have shown that drought increased the accumulation of luteolin in *Ligustrum lucidum* Ait. [37], and quercetin also showed a positive impact on *Vigna unguiculata* L. Walp. [41], but in *Cabernet Sauvignon* [42], quercetin showed no obvious change under water stress. Therefore, further experiments are needed to investigate the effects of abiotic stress on secondary metabolite content in Verbena.

### 3.3. Biological Mechanism of Verbena in Response to Drought Stress

The protective mechanisms of plants in response to stress are regulated by alterations in the expression level of stress-responsive genes. Among the DEGs assigned to KOG and COG, genes related to plants’ stress response accounted for quite a high proportion; 8.50% and 11.00%, respectively. We performed GO and KEGG pathway enrichment analysis and excavated a group of important drought-responsive genes related to multiple biological mechanisms of plant energy production, hormone synthesis, cell signaling, and metabolism. In this study, there are 12, 25 and 24 genes differentially expressed in “glyoxylate and dicarboxylate metabolism”, “citrate cycle (TCA cycle)” and “glycolysis/gluconeogenesis” pathway, respectively, and all of them were up-regulated. This indicates that the energy production of Verbena changed a lot, and the main method of providing ATP was changed from the photosynthetic phosphorylation to the oxidative phosphorylation.

ABA is one of the most important factors in abiotic stress response, and is involved in almost all plant activities, such as photosynthesis, ionic homeostasis, and antioxidant defense [43]. The quantities of gene expression controlling the key enzymes, such as PYL and PP2C in the ABA signaling pathway, have changed a lot. The vicissitudinous DEGs of PYL activate the high expression of PP2C and furthermore promote stomatal closure and decrease transpiration to reduce water loss. Under the “plant hormone signal transduction” pathway, in addition to the enzymes of ABA, the level of genes that control PR1 in the “salicylic acid (SA)”, JAZ and MYC2 in the “jasmonic acid (JA)”, GH3 and SAUR in the “auxin”, and MPK6 together with EBF1_2 in “Ethylene” pathways, were almost all up-regulated (Table 2). This is in line with previous findings that the changes of hormonal synthesis and signal transduction are the conservative mechanisms by which plants response to adverse circumstances. It has been demonstrated that NO (nitric oxide) can act as a signaling molecule to activate ROS-scavenging enzymes in drought stress [44]. Under the pathway of “Nitrogen metabolism”, we observed that the gene expression levels of NR and NirA in the assimilatory nitrate reduction pathway were down-regulated, and the expression of GLT1 and GLUL in the glutamate synthase pathway were up-regulated (Table 6). The glutamate synthase pathway leads to the process of glutamate metabolism and the production of ammonia, and a sufficient amount of oxidized glutamic acid will ensure that there is sufficient carbon skeleton for the tricarboxylic acid cycle to function effectively [45]. Therefore, we can speculate that improving the level of nitrogen metabolism is a very efficient method to help plants resist abiotic stress.

Carbohydrate metabolism is the center of the entire biological metabolism and involves the protein metabolism, lipid metabolism, nucleic acid metabolism and secondary metabolites production. Studies have shown that in rice [46], sugar can be used as a signal to induce the expression of genes associated with abiotic stress. Garg A.K. et al. [47] found that the accumulation of trehalose in rice enabled the transgenic rice to exhibit high salt tolerance, drought tolerance, and low-temperature stress. In this study, there were 28 and 12 DEGs in “Starch and sucrose metabolism” and “Pentose phosphate pathway”, respectively, most of which were up-regulated, which indicates the importance of sugar for Verbena to cope with drought stress. Furthermore, we found a series of pathways for the up-regulation of DEGs expression: protein metabolism such as “Biosynthesis of amino acids”, lipid metabolism such as “Fatty acid metabolism” and “Glycerophospholipid metabolism”, nucleic acid metabolism such as “Pyrimidine metabolism” and “Purine metabolism”, and metabolism of secondary products such as “alpha-Linolenic acid metabolism” and “Flavone and flavonol biosynthesis”. All of these show that carbohydrate metabolism is very important for Verbena to improve its tolerance to adverse environmental conditions.

Moreover, “ubiquitin mediated proteolysis”, an energy-consuming, highly efficient and highly directional protein degradation process, is also noteworthy. It plays an important role in many aspects, such as modulation of the immune and inflammatory responses, the regulation of cell cycle, control of signal transduction pathways, development and differentiation etc. [48,49]. In the present study, there are 16 DEGs in this pathway (Table 7), all of which up-regulate. Half are involved in the regulation of ubiquitin conjugating enzyme, and the others are involved in the regulation of ubiquitin ligase. At present, the understanding of this process is still very limited. The genes participated in this process during drought stress, and the mechanism of the enzymes which were regulated by these genes has yet to be studied.

We found evidence that Verbena responds to drought stress by altering energy synthesis pathways, decreasing transpiration, resetting hormone secretion levels, and increasing cell osmotic pressure and glucose metabolism. In general, Verbena’s defensive response, like most plants under stress conditions, is a process of rebuilding physiologically, biochemically and metabolically, from the growth-oriented to the defensively based one. The genes mentioned in Section 3 are listed in Table 1, Table 2, Table 3, Table 4, Table 5, Table 6 and Table 7 and after counting the TFs to which these genes belong, we constructed the MYB TF phylogenetic tree using the reported abiotic stress genes to prepare for the next work (Figure SA8).

### 3.4. DEGs of Transcription Factors (TFs) under Drought Stress

There are 228 (10.62%) DEGs that were assigned to bHLH TF. The basic helix-loop-helix proteins are one of the largest transcription factor families and are widely distributed in eukaryotes [50]. Many important drought-tolerant genes have been discovered in the bHLH family. Over-expression of OsbHLH148 in the transgenic rice make the plant more drought-tolerant by regulating the jasmonate signal transduction pathway [51]. Up-regulation of bHLH122 can significantly increase ABA levels in cells and it is a positive regulator of drought, NaCl and osmotic signaling [52]. AtbHLH112 is a nuclear-localized protein induced by salt, drought and ABA, and it can increase proline levels and improve ROS scavenging ability to enhance stress tolerance [53]. At the same time, there are also a number of other TFs that play an important role in plants’ resistance to abiotic stresses, such as MYB (9.65%), ERF (8.20%) and NAC (7.13%). In this study, most of the genes that enhance plants’ stress resistance were achieved by overexpression, so we will pay more attention to the up-regulated genes and dig out the role of specific genes in the processes of stress resistance by transgenic and gene-silencing technology.

## 4. Materials and Methods

### 4.1. Plant Materials and Drought Treatments

*Verbena bonariensis* L. with the age of one month were used in this study. The experimental materials were incubated in greenhouse (25 °C, 16 h photoperiod, 50% RH), soil texture for the medium loam, the maximum soil moisture content of 80%. A total of 90 strains of Verbena were randomly divided into two groups T01 (control group) and T02 (drought experiment group), 1 seeding in each pot and 15 in one duplicated group, each of the T01 and T02 contained 3 duplicated groups named T1–T3 and T4–T6, respectively. Determination of soil moisture content was measured by the oven drying method [54]. The soil water content of T01 was kept at 80% of saturated soil moisture all the time, while watering of the T02 was stopped until the soil water content had reduced to 25% of the soil saturated water content. After that, the soil moisture content was measured every 2 days to replenish the amount of deficiency and maintained this drought status for 15 days.

At the 5th, 10th and 15th day, the mature leaves (3rd to 8th functional leaf) were selected randomly from the plants of T01 and T02 for the determination of physiological indexes. After the drought had been maintained for 15 days, samples were collected, rapidly frozen with liquid nitrogen, stored at −80 °C, and finally sent to Biomarker Technologies Co., Ltd. (Beijing, China) for whole-transcriptome sequencing.

### 4.2. Determination of Morphological and Physiological Characters

Vernier calipers were used to measure root length (whichever is longer) and stem length. Chlorophyll content was determined by NanoPhotometer^®^ spectrophotometer (Implen, CA, USA) [55]; Pro content was measured using acidic-ninhydrin method [56]; estimation of soluble protein by Bradford method [57]; nitroblue tetrazolium blue (NBT) reduction method was used to determine the activity of SOD [58]; CAT activity was determined using UV absorption method [59]; POD activity was determined by guaiacol method [60]; determination of MDA content by thiobarbituric acid test (TBA) [61]. Relative water content (RWC) of leaves was measured according to the method of Tambussi E.A. et al. [62] using the formula: RWC = (FW − DW)/(turgid weight − DW) × 100%, where FW is the leaf fresh weight, DW is the leaf dry weight at 85 °C for 3 d, and SW is the turgid weight of leaves after soaking in water for 4 h at room temperature (approximately 25 °C). Physiological measurements were set up for three replicates to reduce the error.

### 4.3. Extraction of RNA, Library Preparation for Transcriptome Sequencing

To ensure the qualified samples were obtained for transcriptome sequencing, total RNA was extracted with Trizol kit (Invitrogen, Carlsbad, CA, USA) and its purity concentration and integrity detected by the Nanodrop, Qubit 2.0, Agilent 2100 method. Then, a total amount of 3 μg qualified RNA per sample was used as input material for the RNA sample preparations. According to manufacturer’s recommendations, sequencing libraries were generated using NEBNext^®^Ultra™ RNA Library Prep Kit for Illumina^®^ (New England Biolabs, Ipswich, MA, USA). The messenger RNAs (mRNAs) were separated from the total RNA by Oligo (dT) and were cleaved into short fragments at random. The first strand cDNA was synthesized by random hexamer primer, then the buffer, dNTPs, DNA polymerase I and RNase H were used to synthesize the second strand cDNAs. Lastly, the cDNAs were purified with AMPure XP beads and after end-repair and single nucleotide A (adenine) addition, the qualified cDNA libraries were constructed by PCR enrichment. After the cDNA libraries were constructed, Qubit 2.0 was used for preliminary quantification, and then the Agilent 2100 was used to detect the insert size of the libraries. After that, the Q-PCR method was used to accurately quantify the effective concentration of the libraries (effective library concentration > 2 nM) to ensure library quality. After passing the screening, high-throughput sequencing was performed with Illumina Hiseq Xten. The raw sequencing data have been submitted to the National Center for Biotechnology Information Search database (NCBI) Sequence Read Archive database with accession number SRP132610.

### 4.4. Transcriptome Assembly and Gene Functional Annotation

A total of 44.59 Gb of clean data was removed from the original data, including low-quality data. Then Trinity software was used to break reads into shorter K-mers, extend them to obtain Contigs, gather a collection of Contig clusters, and finally obtain the transcript sequence by using de Bruijin graphs algorithm method and reads.

Using BLAST software, the sequencing of the Unigene sequence was compared with NR, KOG, COG, GO, KEGG, Pfam and Swissprot-Annotation databases. After the prediction of the amino acid sequence of the unigene, HMMER software [63] was used to compare it with the Pfam database [64] to obtain functional annotation information of all unigenes.

### 4.5. Differential Expression Analysis

Differential expression analysis of the sample groups was performed using DESeq [65] to obtain a set of DEGs between the drought group and the control group, and the False Discovery Rate (FDR) was used to correct the *p*-value of the multiple-hypothesis test. In this study, FDR < 0.01 and FC (Fold Change) ≥ 4 were set as the threshold for significantly differential expression, and could be used to prepare for the follow-up study on the drought stress of Verbena.

### 4.6. Quantitative Real-Time PCR Analysis

In order to clarify the stress response variation tendency of Verbena at the transcriptional level on different stages of drought stress, we selected 10 DEGs to participate in different important biological processes for qRT-PCR with SsoFast™ EvaGreen^®^ Supermix every 5 days. A 20 μL fluorescent quantitative reaction system contains 10 μL of stain, 2 μL of cDNA template and 300 nM of primers. The PCR settings are as follows:

TemperatureTimeCycle95 °C30 s
95 °C15 s40 cycles60 °C30 s

Relative expression levels were calculated by the 2^−ΔΔ*C*t^ method, and a β-actin gene of *Verbena bonariensis* (Forward primer: GAAAGATGGCTGGAAGAGGG, Reverse primer: GCTATGAACTCCCTGATGGTC) was used as the reference for quantitative expression analysis. The expression pattern of DEGs was analyzed by melting furnace curve. The qRT-PCR primers are shown in Table SA6.

## 5. Conclusions

The development of plant genomics plays an important role in the effective use of modern molecular biology methods for the genetic improvement of species. After sequencing the transcriptome of drought-stressed Verbena materials by high-throughput sequencing and verifying the results by qRT-PCR, we finally identified DEGs and TFs related to stress resistance and analyzed their biomodulation mechanism, and this proved Verbena’s commendable drought tolerance at physiological and molecular levels. These data will provide excellent genetic resources for improving the drought tolerance of crops and lay a good foundation for the follow-up study of Verbena.

## Figures and Tables

**Table 1:** DEGs (analysis of differentially expressed genes) in “Porphyrin and chlorophyll metabolism” pathway of KEGG (Kyoto Encyclopedia of Genes and Genomes).

Term	Gene ID	log2FC	Gene Description	FDR
UROD	c76376.graph_c0	2.33	uroporphyrinogen decarboxylase chloroplast precursor	1.44 × 10^−5^
COX15	c59080.graph_c0	2.40	uroporphyrinogen decarboxylase chloroplast precursor	2.71 × 10^−8^
FECH	c69481.graph_c0	2.58	protoporphyrin/coproporphyrin ferrochelatase	7.56 × 10^−5^
	c86128.graph_c2	3.02	chloroplastic isoform X2	1.01 × 10^−7^
EARS	c69469.graph_c0	2.43	glutamyl-tRNA reductase	1.14 × 10^−6^
	c72758.graph_c0	2.31	Porphyrin and chlorophyll metabolism	2.72 × 10^−3^
hemA	c85183.graph_c0	4.04	glutamyl-tRNA reductase 1, chloroplastic-like	1.98 × 10^−11^
	c77400.graph_c0	−2.69	hypothetical protein	9.98 × 10^−38^
	c77400.graph_c1	−2.65	glutamyl-tRNA reductase 1, chloroplastic	1.84 × 10^−31^
	c77400.graph_c2	−2.68	glutamyl-tRNA reductase 1, chloroplastic	1.67 × 10^−23^
chlH	c88820.graph_c1	−2.50	magnesium chelatase subunit H	1.91 × 10^−60^
chlE	c77176.graph_c0	−2.24	magnesium-protoporphyrin IX monomethyl ester (oxidative) cyclase	7.54 × 10^−37^
por	c85861.graph_c0	−3.48	protochlorophyllide reductase	1.36 × 10^−11^
chlP	c80298.graph_c1	−2.94	geranylgeranyl diphosphate/geranylgeranyl-bacteriochlorophyllide a reductase	1.13 × 10^−47^

**Table 2:** DEGs related to hormone synthesis in response to drought stress.

Term		Gene ID	log2FC	Gene Description	FDR
ABA	PYL/PYR	c72499.graph_c2	5.61	abscisic acid receptor PYR/PYL family (A)	5.09 × 10^−14^
		c64811.graph_c0	−2.31	abscisic acid receptor PYR/PYL family (A)	2.69 × 10^−58^
		c73702.graph_c1	−2.28	K14496 abscisic acid receptor PYR/PYL family (A)	0.00000743
	PP2C	c86830.graph_c0	2.60	probable protein phosphatase 2C 51	1.62 × 10^−19^
SA	PR1	c31398.graph_c0	4.97	basic form of pathogenesis-related protein 1-like	2.11 × 10^−159^
JA	JAZ	c75424.graph_c0	2.43	protein TIFY 10B-like	4.90 × 10^−58^
		c75566.graph_c0	2.14	jasmonate ZIM domain-containing protein (A)	8.12 × 10^−44^
		c77115.graph_c1	3.17	jasmonate ZIM domain-containing protein (A)	2.58 × 10^−74^
		c77115.graph_c2	3.22	Protein TIFY 10B	2.80 × 10^−87^
		c88229.graph_c0	2.13	protein TIFY 9-like	0.000018
	MYC2	c88848.graph_c1	2.14	transcription factor MYC2-like	1.13 × 10^−24^
Auxin	GH3	c78593.graph_c1	2.24	auxin responsive GH3 gene family (A)	5.93 × 10^−6^
		c83994.graph_c0	3.57	auxin responsive GH3 gene family (A)	3.63 × 10^−62^
	SAUR	c76579.graph_c0	2.39	uncharacterized protein	3.12 × 10^−16^
		c80406.graph_c5	2.71	hypothetical protein MIMGU_mgv1a0212152mg	4.51 × 10^−26^
		c63583.graph_c0	−4.29	auxin-induced protein 10A5	1.74 × 10^−17^
		c64412.graph_c0	−2.18	SAUR family protein (A)	5.04 × 10^−9^
		c65963.graph_c0	−3.66	indole-3-acetic acid-induced protein ARG7-like	1.28 × 10^−13^
		c84555.graph_c1	−4.21	SAUR family protein (A)	4.31 × 10^−11^
Ethyle-ne	MPK6	c75482.graph_c0	2.19	mitogen-activated protein kinase 8	1.2624 × 10^−3^
	EBF1/2	c70061.graph_c0	2.28	EIN3-binding F-box protein (A)	4.3002 × 10^−3^

**Table 3:** DEGs in “Photosynthesis-antenna Proteins” pathway of KEGG.

Term	Gene ID	log2FC	Gene Description	FDR
LHCA1	c75167.graph_c0	−2.30	chlorophyll a-b binding protein 6, chloroplastic	1.13 × 10^−47^
LHCA2	c57238.graph_c0	−2.30	chlorophyll a-b binding protein, chloroplastic	1.52 × 10^−37^
LHCA3	c71085.graph_c0	−2.07	chlorophyll a-b binding protein 8, chloroplastic-like	3.11 × 10^−14^
	c85515.graph_c0	−2.33	chlorophyll a-b binding protein 8, chloroplastic	2.41 × 10^−138^
	c71085.graph_c1	−2.20	chlorophyll a-b binding protein 8, chloroplastic-like	5.63 × 10^−5^
LHCA4	c57961.graph_c0	−3.78	chlorophyll a-b binding protein 4, chloroplastic	3.42 × 10^−11^
	c81195.graph_c1	−3.42	agamous-like MADS-box protein AGL21 isoform X3	1.69 × 10^−77^
	c31746.graph_c0	−3.92	chlorophyll a-b binding protein P4, chloroplastic-like	1.20 × 10^−120^
LHCB1	c85665.graph_c1	−3.25	chlorophyll a/b-binding protein PS II-Type I	6.78 × 10^−29^
	c85665.graph_c2	−3.68	chlorophyll a-b binding protein 21, chloroplastic-like	6.40 × 10^−63^
	c83506.graph_c0	−3.53	chlorophyll a/b-binding protein, partial	1.02 × 10^−12^
LHCB2	c31726.graph_c0	−2.58	chlorophyll a-b binding protein 5, chloroplastic	1.26 × 10^−46^
	c77073.graph_c0	−3.51	chlorophyll A/B binding protein, putative	7.67 × 10^−58^
LHCB3	c82382.graph_c0	−2.60	chlorophyll a-b binding protein 13, chloroplastic	5.22 × 10^−47^
	c82382.graph_c1	−2.74	chlorophyll a-b binding protein 13, chloroplastic	5.88 × 10^−32^
	c84778.graph_c0	−2.35	chlorophyll a-b binding protein 13, chloroplastic	1.14 × 10^−20^
LHCB4	c57394.graph_c0	−3.66	chlorophyll a-b binding protein CP29.1, chloroplastic	4.63 × 10^−60^
LHCB5	c72073.graph_c1	−2.03	chlorophyll a-b binding protein CP26, chloroplastic	4.96 × 10^−43^
	c72073.graph_c0	−2.25	chlorophyll a-b binding protein CP26, chloroplastic	7.71 × 10^−40^
LHCB6	c76630.graph_c1	−2.38	hypothetical protein MIMGU_mgv1a012260mg	2.07 × 10^−23^

**Table 4:** DEGs in “Betalain biosynthesis” pathway of KEGG.

Term	Gene ID	log2FC	Gene Description	FDR
TYR	c83086.graph_c0	2.69	Tyrosinase	2.09 × 10^−^^5^
COMT	c26366.graph_c0	3.02	catechol *O*-methyltransferase	7.64 × 10^−^^5^
DOPA	c75132.graph_c0	3.38	PREDICTED: 4,5-DOPA dioxygenase extradiol-like	1.87 × 10^−^^89^

**Table 5:** DEGs in “Flavone and flavonol biosynthese” pathway of KEGG.

Term	Gene ID	log2FC	Gene Description	FDR
E1.14.13.21	c32062.graph_c0	2.11	benzoate 4-monooxygenase cytochrome P450	1.06 × 10^−^^5^
AOMT	c57467.graph_c0	4.20	PREDICTED: flavonoid 3&apos; 5&apos; -methyltransferase-like	1.59 × 10^−^^13^
	c67675.graph_c0	3.17	PREDICTED: flavonoid 3&apos; 5&apos; -methyltransferase-like	2.84 × 10^−^^22^
C12RT1	c69454.graph_c0	4.05	hypothetical protein MIMGU_mgv1a022315mg	1.81 × 10^−^^17^

**Table 6:** DEGs in “Nitrogen metabolism” pathway of KEGG.

Term	Gene ID	log2FC	Gene Description	FDR
NR	c88329.graph_c0	−2.65	Nitrate reductase 2	1.32 × 10^−97^
NirA	c85021.graph_c0	−2.64	Ferredoxin–nitrite reductase	4.29 × 10^−84^
GLUL	c89561.graph_c0	2.21	glutamine synthetase4	2.24 × 10^−^^7^
GLT1	c85092.graph_c2	2.29	glutamate synthase (NADPH/NADH)	1.53 × 10^−^^5^

**Table 7:** DEGs in “Ubiquitin mediated proteolysis” pathway of KEGG.

Term		Gene ID	log2FC	Gene Description	FDR
E2	UBE2A	c56569.graph_c0	2.73	ubiquitin-conjugating enzyme E2 A	9.53 × 10^−7^
	UBE2O	c78080.graph_c1	2.32	ubiquitin-conjugating enzyme E2 O; A orthologs to drought gene GmMYB177	1.03 × 10^−6^
	UBE2W	c43734.graph_c0	3.58	ubiquitin-conjugating enzyme E2 W	3.64 × 10^−6^
	UBE2N	c61661.graph_c0	2.30	ubiquitin-conjugating enzyme E2 N	3.35 × 10^−6^
	UBE2D-E	c89609.graph_c0	2.54	ubiquitin-conjugating enzyme E2 D/E	4.15 × 10^−6^
	UBE2I	c46599.graph_c0	2.71	ubiquitin-conjugating enzyme E2 I; A orthologs to drought gene GmMYB177	3.46 × 10^−8^
	UBE2G1	c26287.graph_c0	2.14	ubiquitin-conjugating enzyme E2 G1	2.449 × 10^−3^
		c25902.graph_c0	3.11	ubiquitin-conjugating enzyme E2 G1	1.23 × 10^−12^
E3	ARF-BP1	c84837.graph_c1	2.40	E3 ubiquitin-protein ligase HUWE1	2.58614 × 10^−4^
	UBE4B	c71025.graph_c0	2.32	ubiquitin conjugation factor E4 B	1.45681 × 10^−4^
	CYC4	c75600.graph_c0	2.46	peptidyl-prolyl cis-trans isomerase-like 2	1.46 × 10^−5^
	PRP19	c60805.graph_c0	2.06	pre-mRNA-processing factor 19	2.56 × 10^−5^
	Cul3	c63726.graph_c0	2.00	cullin 3 (A)	2.73 × 10^−5^
	CYC4	c75600.graph_c0	2.46	peptidyl-prolyl cis-trans isomerase-like 2	1.46 × 10^−5^
	SYVN	c79541.graph_c0	2.01	ubiquitin-protein ligase synoviolin	1.091839 × 10^−3^
	Cdh1	c47817.graph_c0	2.93	cell division cycle 20-like protein 1, cofactor of APC complex (A)	1.53 × 10^−5^
	TRIP12	c82561.graph_c0	2.52	E3 ubiquitin-protein ligase TRIP12	2.08 × 10^−9^

## Supplementary Materials

The following are available online at http://www.mdpi.com/1422-0067/19/6/1751/s1.
