# Supplementary material for: Whole-Transcriptome Sequence Analysis of Verbena bonariensis in Response to Drought Stress
Source: Int J Mol Sci. 2018 Jun 13;19(6):1751. doi: 10.3390/ijms19061751 (PMC6032440; doi:10.3390/ijms19061751)
Supplement: Supplementary file 1 [file ijms-19-01751-s001.pdf]

Table SA1. Assessmented statistics of six samples' sequencing data.

Samples		Read Number	Base Number	GC Content	%≥Q30
Control group 1	T01	25,189,726	7545,832,736	44.91%	92.87%
Control group 2	T02	23,985,897	7186,098,010	44.95%	92.95%
Control group 3	T03	25,666,923	7682,907,212	44.72%	92.97%
experimental group 1	T04	24,253,957	7261,480,372	44.43%	93.02%
experimental group 2	T05	25,483,372	7631,598,048	44.41%	93.07%
experimental group 3	T06	24,318,468	7283,574,304	44.46%	93.10%

Table SA2. Length distribution of the transcripts and unigenes clustered from *de novo* assembly.

Length range	Transcript	Unigene
200-300	57,750(22.36%)	44,039(39.56%)
300-500	45,715(17.70%)	26,934(24.20%)
500-1000	51,288(19.85%)	19,318(17.35%)
1000-2000	56,712(21.95%)	12,826(11.25%)
2000^{+}	46,861(18.14%)	8,196(7.36%)
Total number	258,32	111,313
Total length	294,409,787	77,594,018
N50 length	1,951	1,223
Mean length	1,139.683141	697.0795684

Table SA3. Annotationed unigenes and DEGs of *Verbena bonariensis.*

Term	Unigene	DEGs
Total annotated	53,757	4,165
NR	51,325	4,155
KOG	28,994	2,426
COG	14,836	1,554
GO	20,988	1,756
KEGG	16,938	1,521
Swiss-Prot	27,990	2,577
eggNOG	47,283	3,957
Pfam	32,735	3,419

Table SA4. The top five significantly enriched nodes of BP, MF and CC in GO..

Term	GO. ID	Term	DEGs in term	All genes in term	FDR
BP	GO:0015074	DNA integration	4	415	1.70E-12
	GO:0006278	RNA-dependent DNA replication	6	301	6.70E-11
	GO:0009765	photosynthesis, light harvesting	17	31	2.70E-08
	GO:0043581	mycelium development	18	68	1.10E-06
	GO:0006099	tricarboxylic acid cycle	11	124	1.40E-05
MF	GO:0003676	nucleic acid binding	209	3,521	1.80E-12
	GO:0003964	RNA-directed DNA polymerase activity	6	317	2.30E-10
	GO:0016168	chlorophyll binding	14	23	7.90E-07
	GO:0003735	structural constituent of ribosome	49	683	6.10E-05
	GO:0016717	oxidoreductase activity etc.	6	36	0.00016
CC	GO:0000329	fungal-type vacuole membrane	5	15	1.80E-05
	GO:0005840	ribosome	63	807	1.80E-05
	GO:0005634	nucleus	128	2,181	6.60E-05
	GO:0009523	photosystem II	18	37	8.30E-05
	GO:0009522	photosystem I	14	33	0.00014

Table SA5. The top ten significantly enriched pathways of DEGs in KEGG.

KEGG. ID	Pathway	DEGs in pathway	All genes in pathway	FDR
ko00196	Photosynthesis - antenna proteins	20	31	7.90E-14
ko00909	Sesquiterpenoid and triterpenoid biosynthesis	11	31	7.12E-05
ko00195	Photosynthesis	16	64	0.000217806
ko04626	Plant-pathogen interaction	52	344	0.000394935
ko00944	Flavone and flavonol biosynthesis	4	9	0.00671946
ko00965	Betalain biosynthesis	3	5	0.007224872
ko00592	alpha-Linolenic acid metabolism	16	88	0.007560261
ko01212	Fatty acid metabolism	29	200	0.012548266
ko00860	Porphyrin and chlorophyll metabolism	14	79	0.014940178
ko00785	Lipoic acid metabolism	4	12	0.020988586

Table SA6. Primers of qRT-PCR for validation of the selected DEGs.

Gene ID	Description	Primers
c74964.graph_c0	Energy production and conversion	F：GGAGAGGAGAGAGAGAGAAATGTGG<br>R：ATGAACTGGCAAACAAACATGGAAG
c77073.graph_c0	light-harvesting complex II chlorophyll a/b binding protein 2（ LHCB2）	F：ACACCAAGTCCAACAACATCACATAGAAG<br>R：TCGGCGGTTACAACTTACAATGTTATTT
c89561.graph_c0	glutamine synthetase（GLUL）	F：TTGCTCTGATATCGCTAGTCAGATAACC<br>R：CAATCAGCTATACATTCATTCACTTTCAA
c86830.graph_c0	protein phosphatase 2C（PP2C）	F：TTTATTCATGCAGCGTTTCTCTCGTATC<br>R：TATGACAGGGTTTTTTTAGCTCATTTCTCA
c75482.graph_c0	mitogen-activated protein kinase 6（MPK6）	F：AAAATTTCTTGCCTTAACATGAAGATTTAA<br>R：CAACAAATAACCAGACTCAAGAGGGAGTT
c83922.graph_c0	bHLH transcription factor	F：GTTAAAAATTTCTTGCCTTAACATGAAG<br>R：CTGAATCATAATGACACGTAAAATCCA
c73643.graph_c1	Lipid transport and metabolism	F：CTTTCACAATGGCAGGTTTATACAGAG<br>R：GTATTCAACTGGTGGAGCTCTGTAGTAAG
c87759.graph_c0	Carbohydrate transport and metabolism	F：AAGCTTTCACAATGGCAGGTTTATACAG<br>R：CTCTCTGTGTATCATCGATCATCATATTTA
c56670.graph_c0	C2H2 transcription factor	F：GATGATGATGATGATGATGATGCTAATG<br>R：TTATATTTCTTTTAGGGACATCAAACATG
c82194.graph_c0	Function unknown	F：GATGATGATGATGATGATGATGCTAATG<br>R：TACTCTTGTCTCTTGCTGCTTATGATTAT

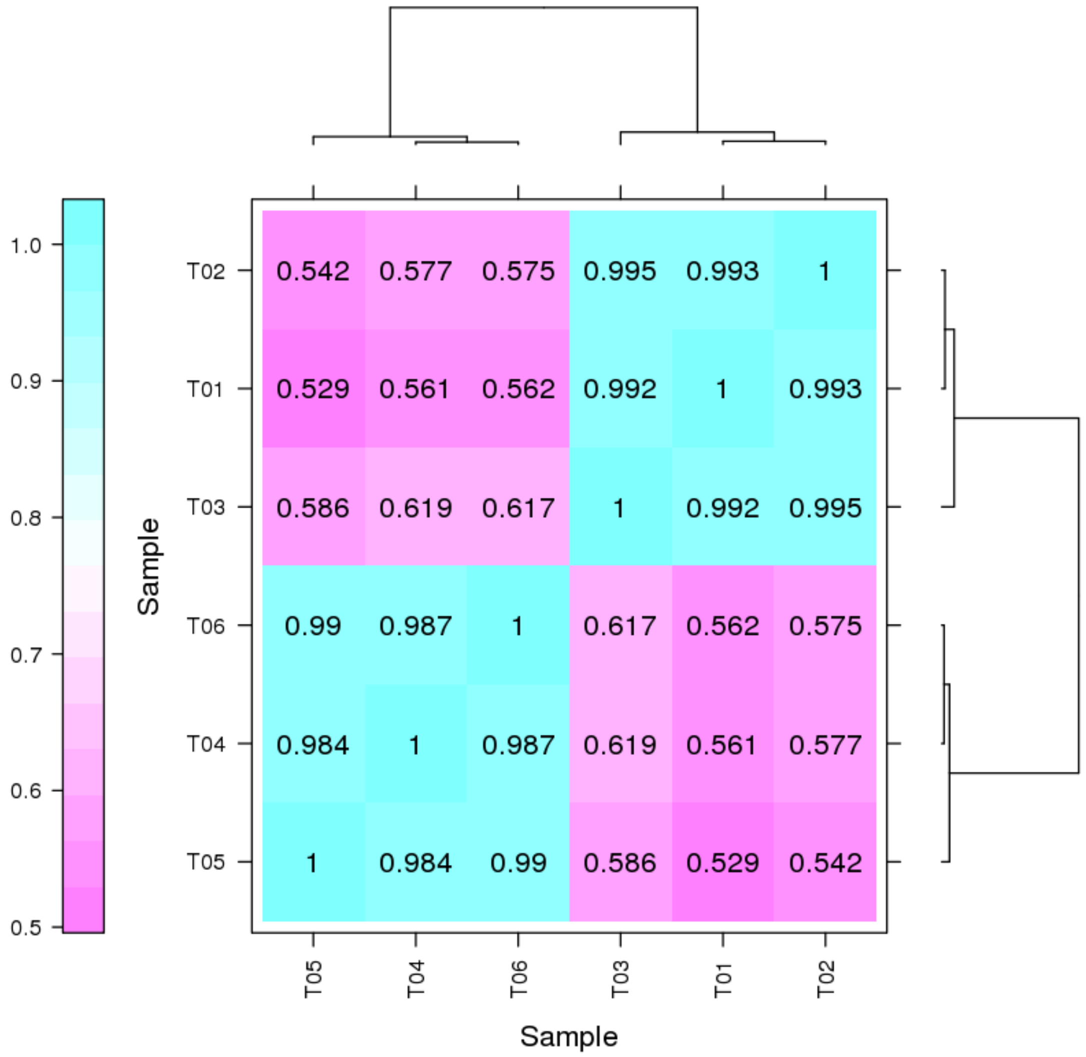

Figure SA1. The Pearson' s Correlation Coeffiencient *r* between control and experiment samples. In this figure, different columns represent different samples, and different rows represent different genes. The color represents the base 2 logarithm of the FPKM expression level of the gene in the sample. Color from red to green means the correlation gradually increased. Clustering together with similar correlations.

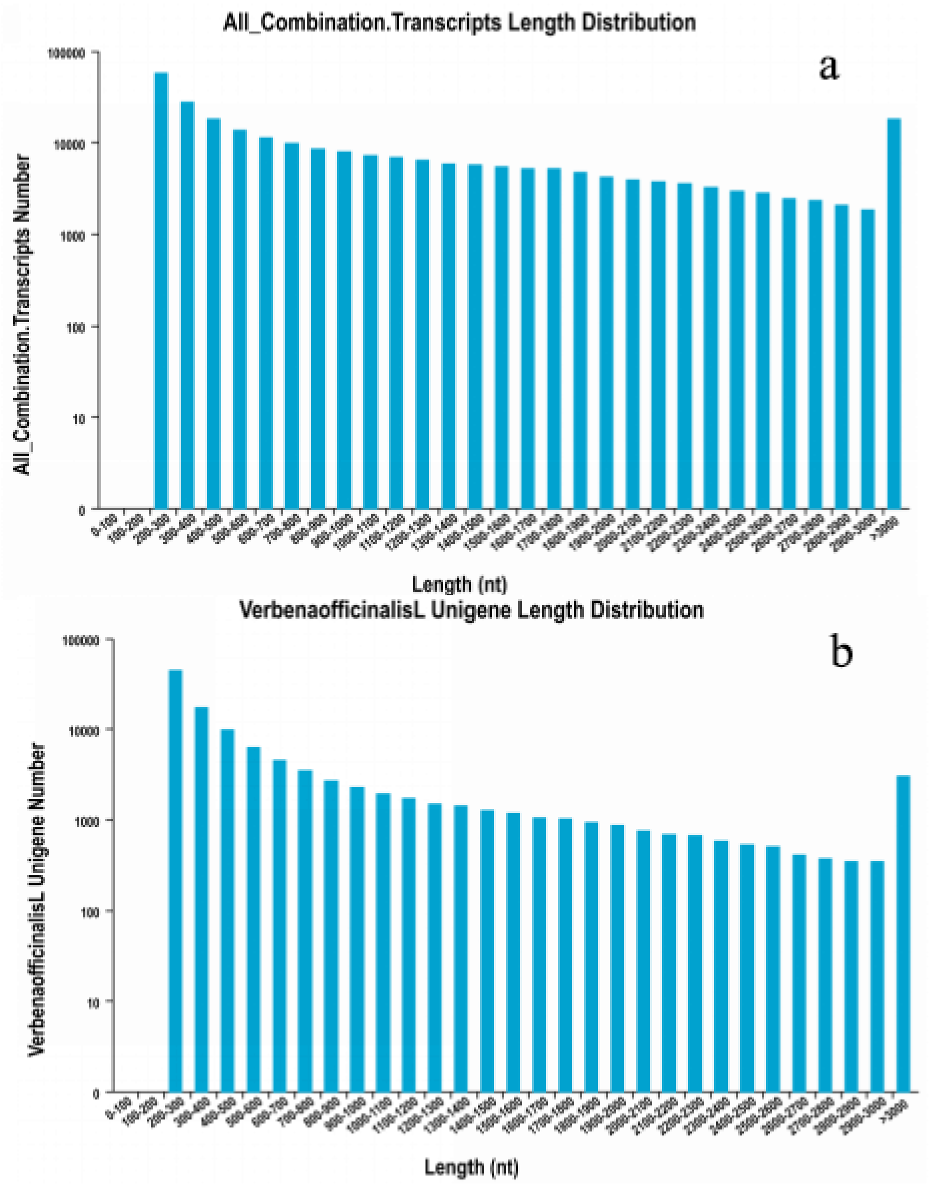

Figure SA2. **(a)** Length distribution of Phormium tenax transcripts. **(b)** Size distribution of Phormium tenax unigenes.

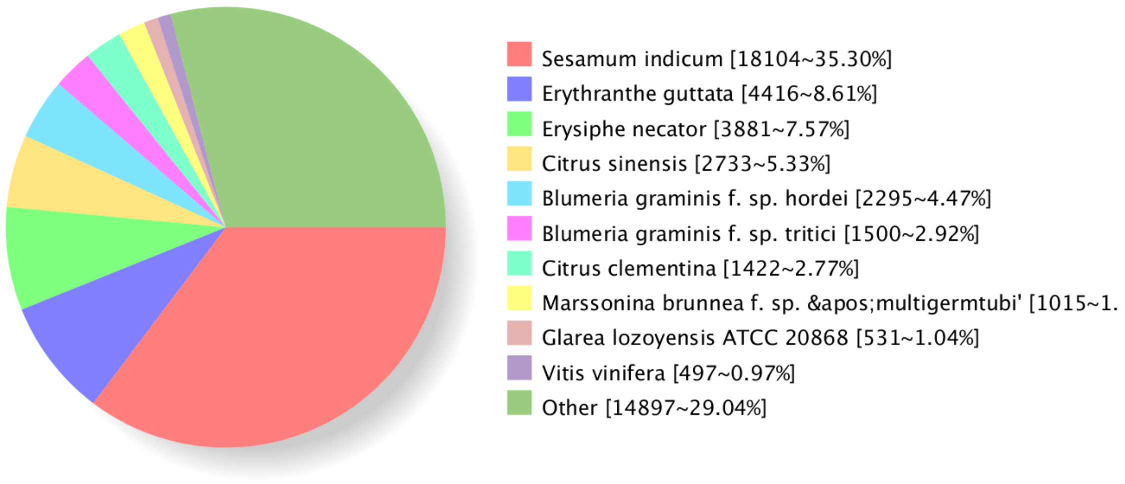

Figure A3. Nr homologous species distribution.

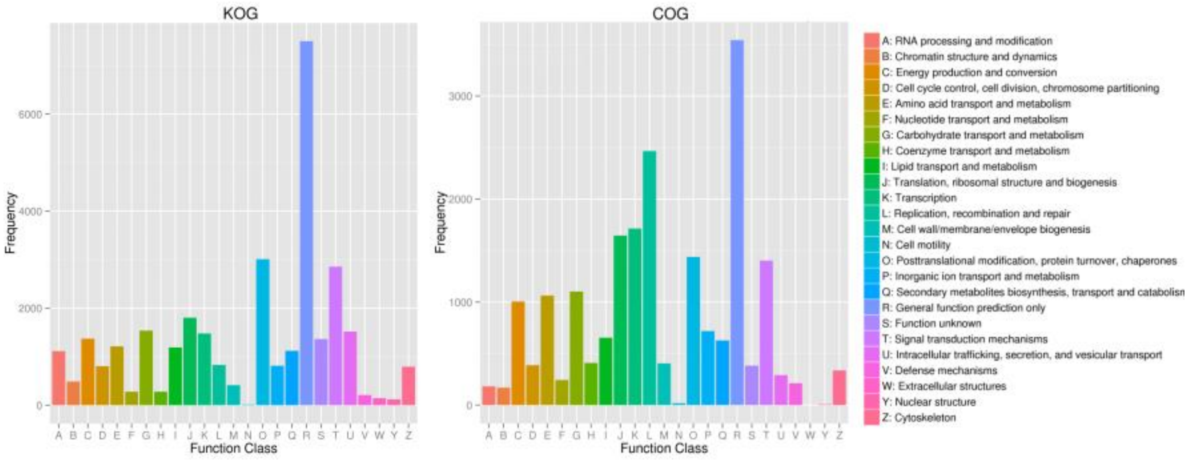

Figure SA4. KOG and COG function Classification of unigenes. The abscissa is the function classification of the database and the ordinate is the number of unigenes annotated in it.

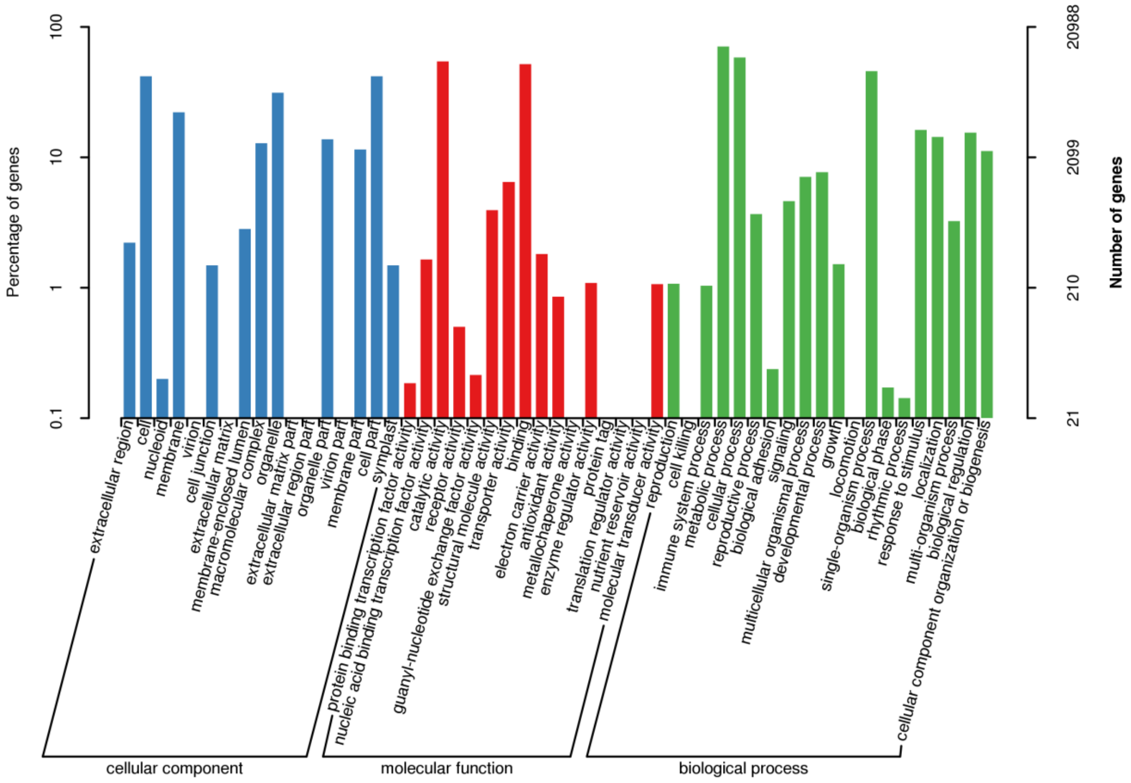

Figure SA5. GO classification of unigenes. The ordinate in the left represents the percentage of the number of genes, the right ordinate represents the number of unigenes. The abscissa is the classification of GO.

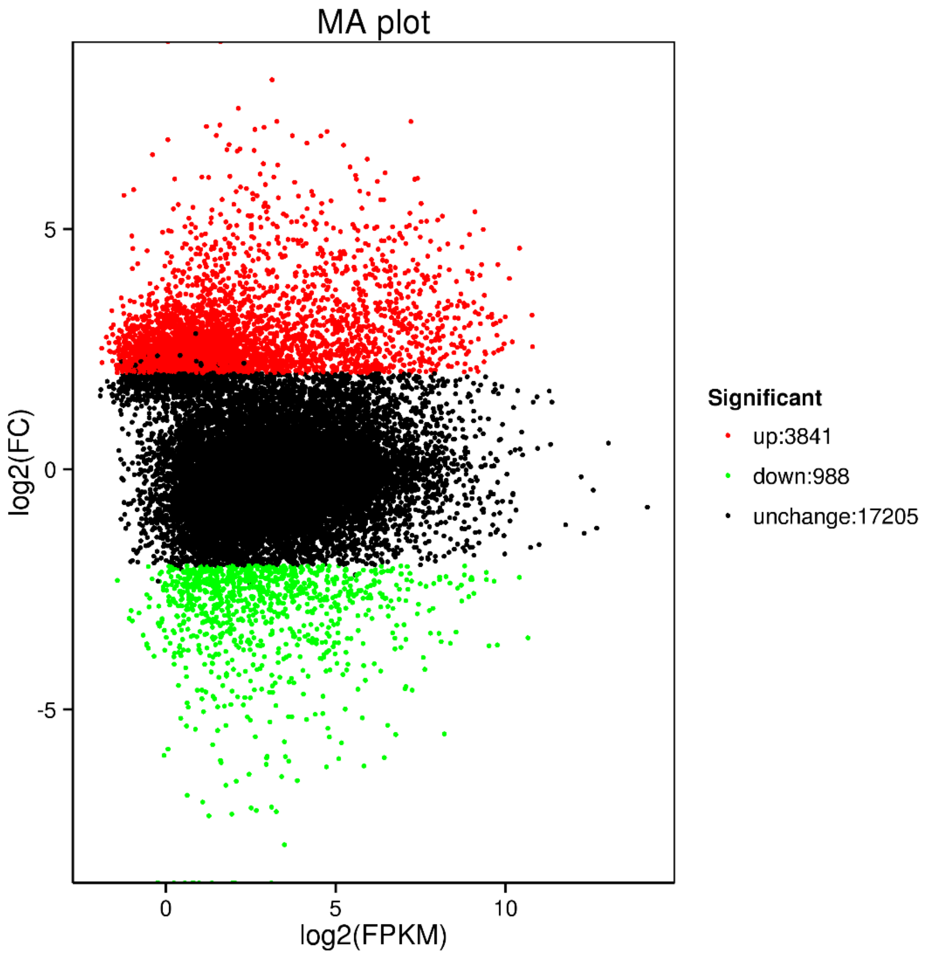

Figure SA6. Volcano map of DEGs. The abscissa is the value of log_2 (FPKM). The ordinate is the value of log_2FC which means the difference of the expression level. Each point in the figure represents a gene, the green dots represent the down-regulation of gene expression and red dots represent the up-regulation of genes. Black dots represent no significant difference between the expression of the gene.

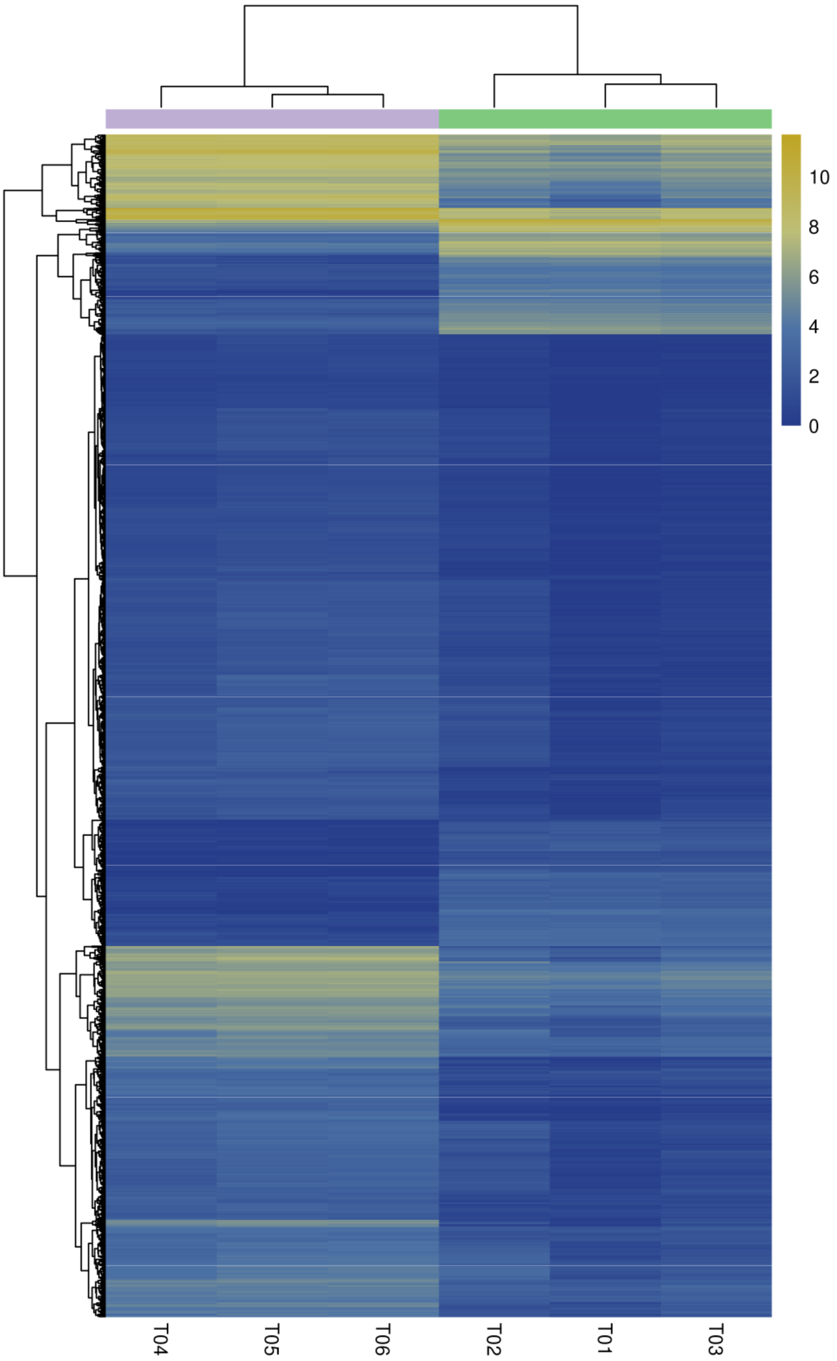

Figure SA7. The expression of all differentially expressed genes was clustered. The abscissa is the gene and the ordinate is the sample. Color represents the level of expression, From blue to yellow on behalf of the expression value from high to low.

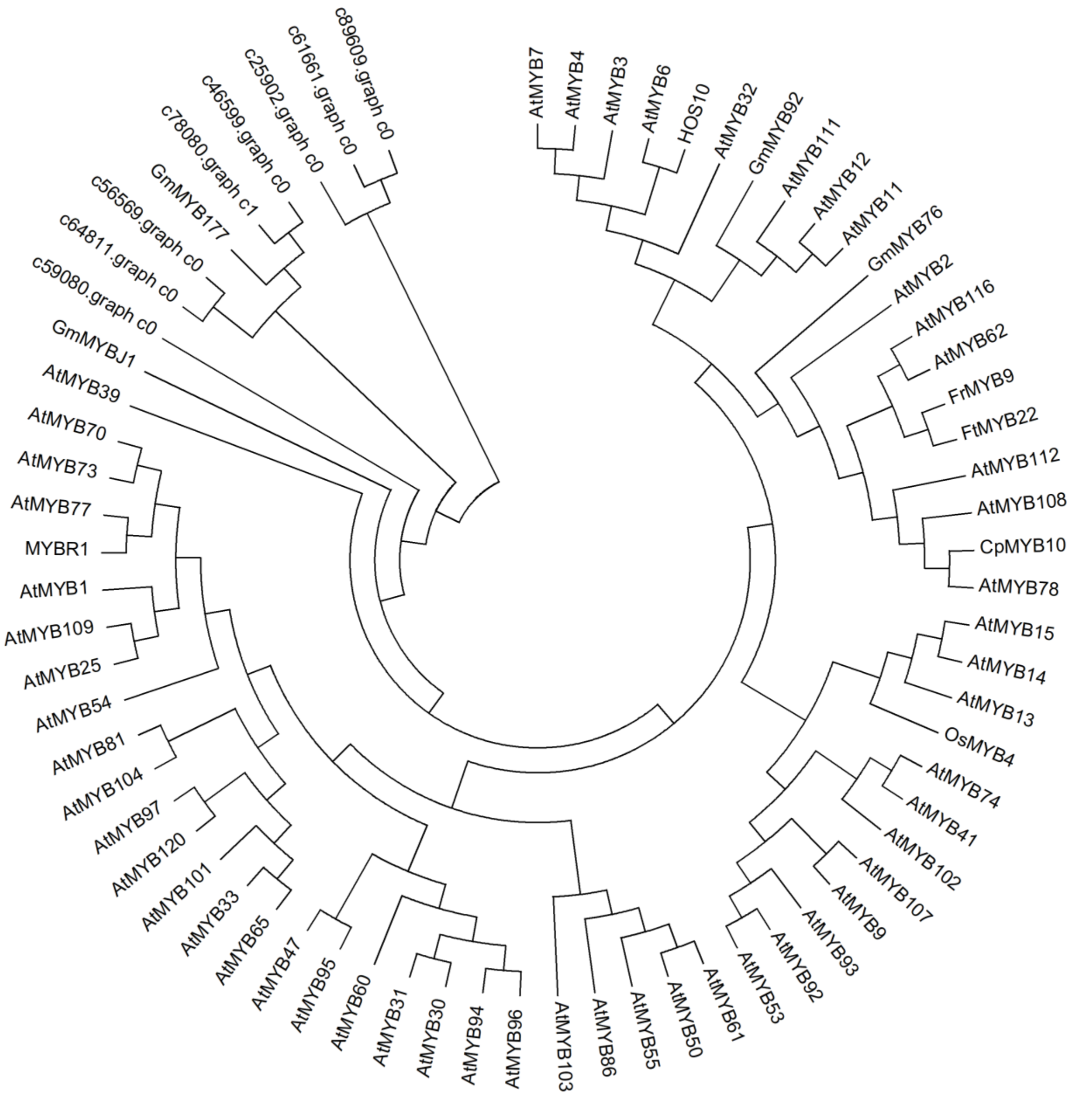

Figure SA8. The phylogenetic tree of MYB TF.
